# Infertility in the Global South: Raising awareness and generating insights for policy and practice

**Published:** 2017-03

**Authors:** T Gerrits, F Van Rooij, T Esho, W Ndegwa, J Goossens, A Bilajbegovic, A Jansen, B Kioko, L Koppen, S Kemunto Migiro, S Mwenda, H Bos

**Affiliations:** University of Amsterdam, Amsterdam Institute of Social Science Research; University of Amsterdam, Research Institute Child Development and Education; Technical University of Kenya, Department of Community and Public Health; Footsteps for Fertility Foundation, Nairobi, Kenya; The Walking Egg, Genk, Belgium

**Keywords:** Advocacy, Ghana, infertility, IVF, Kenya, male involvement, more affordable treatment, prevention, reproductive rights, stigmatization, support groups

## Abstract

Infertility is a highly prevalent reproductive health condition in the global South, which often has a devastating impact on the people concerned. Yet, thus far it hardly received any attention from policy makers, Non-Governmental Organizations (NGOs) or donors working in the field of Sexual and Reproductive Health and Rights (SRHR). For this reason we have set up a project to increase knowledge and awareness about infertility and childlessness among those stakeholders and organizations and to generate insight into (possible) interventions in this field. The project received a grant by Share-Net International (the Knowledge Platform in the field of SRHR, funded by the Dutch Ministry of Foreign Affairs) and is a unique collaboration between universities, fertility clinics, fertility support groups and the Walking Egg Foundation. The project consists of multimethods studies in Ghana and Kenya as well as dissemination workshops and meetings in these countries and the Netherlands. The first workshops in Kenya have already taken place with successful feedback from stakeholders. In this commentary we provide insight into the project and the main points and recommendations discussed in the Workshops in Kenya.

## Infertility in the Global South

Infertility is a highly prevalent reproductive health condition, globally affecting 15 % of couples of the reproductive-age population. In certain areas - in Central and Southern Africa - the percentage is even higher due to a high rate of sexually transmitted infections (STIs), post-abortive complications, HIV/ AIDS (Human Immunodeficiency Virus Acquired Immune Deficiency Syndrome), postpartum and iatrogenic infections (Vayena et al., 2002). Infertility and involuntary childlessness often have a devastating impact on the people concerned, in particular in non-western settings where parenthood is culturally mandatory (Van Balen and Gerrits, 2001; Inhorn and Van Balen, 2002; SekaddeKigondu et al., 2003; Dhont et al., 2011; Dyer, 2007, 2012; Donkor, 2008; Inhorn, 2009; Van Balen and Bos, 2009; NdegWa, 2014).

**Figure 1:**
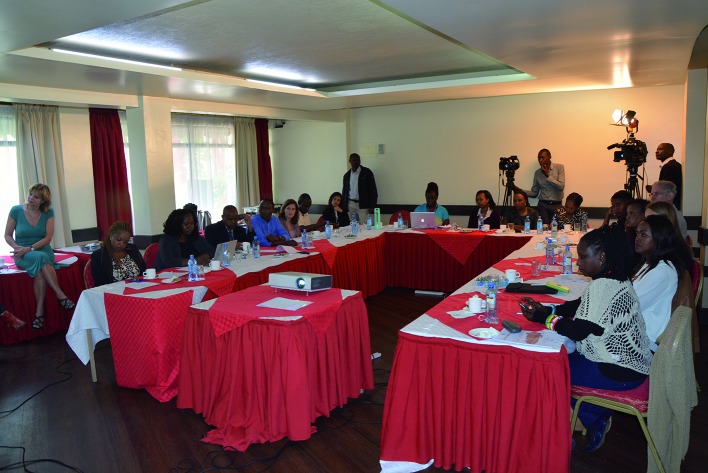
— Workshop with practitioners and press.

Infertility strongly impacts on the emotional impact at a personal level (grief and depression), but also on marital relationships resulting in disrespectful treatment by husbands and/or in-laws, domestic violence, abandonment, divorce and/or polygamy (Inhorn and Van Balen, 2002; SekaddeKigondu et al., 2003; Dyer, 2007, 2012; Donkor, 2008; Van Balen and Bos, 2009; Dhont et al., 2011). Infertility may also lead to health risks given people with fertility problems are at greater risk of exposure to HIV and other STIs as a result of attempts to conceive extramaritally (Dhont et al., 2010). At community level, childless women and men may suffer from social exclusion and stigmatization. Additionally, their social security might be affected as they do not have children to take care of them at old age or when sick. Finally, infertility often leads to economic hardship, as childless couples often spent their incomes on traditional and/or biomedical treatments (Van Balen and Gerrits, 2001; Dyer, 2012; Inhorn and Patrizio, 2014;). Given the severity and diversity of these effects, infertility is a condition that affects basic human rights of individuals. Therefore, reproductive rights must include the right to address fertility problems (Inhorn and Van Balen, 2002; Inhorn, 2009).

While at various international fora and in various declarations (United Nations, 2004; Ombelet, 2012) infertility is indeed recognized as a major reproductive health concern, until recently this recognition has barely led to the development of concrete interventions aimed at addressing infertility in resource poor areas (Ombelet, 2012; Inhorn and Patrizio, 2015). Hardly any initiatives have been undertaken, neither to inform and support infertile people or decrease their stigmatization, nor to prevent infertility. In addition, diagnostic examinations and potentially effective and nonexpensive low tech treatments are seldom offered in a systematic way in the public health system and high tech treatments (like IVF) are only offered in private clinics at high costs and are thus inaccessible for most people (Vayena et al., 2002; Osei, 2014).

Reasons given for the neglect of infertility in the global south are concerns about population growth (rather than infertility), scarcity of health care resources and infrastructure and the heavy burden of other life-threatening diseases such as HIV/ AIDS and maternal mortality; yet, from a reproductive rights perspective these reasons are highly contested (Van Balen and Gerrits, 2001; Inhorn, 2009; Ombelet, 2012). Further, it has been argued that lack of knowledge and awareness among national policy makers, NGOs and donors regarding the severity of the problem of infertility reinforces the notion that infertility is not a priority reproductive health concern, especially in African countries (Etuk, 2009; Oluyemi and Truter, 2011; Nahar, 2012). The programmatic neglect of infertility in the field of SRHR formed the major reason to set up a project that is focusing on infertility and fertility care in Kenya and Ghana.

## The project

The title of this project is “Women and Men with Fertility Problems: A Quantitative and Qualitative Study on a New IVF Treatment and Fertility Support Groups in Kenya and Ghana”. The project was carried out with a small grant from Share-Net International, which is the Knowledge Platform for Sexual and Reproductive Health and Rights (SRHR), initiated and funded by the Dutch Ministry of Foreign Affairs. This (research) project, aims to generate new insights about the views, needs and experiences regarding infertility and fertility care and to increase knowledge and awareness about infertility, childlessness and possible interventions among relevant stakeholders and institutions in Kenya and Ghana.

The uniqueness of the project was not only the focus, but also the collaboration between researchers from different disciplinary backgrounds, such as anthropology, child development and education, and public health (as a consequence also different research methods were used in the project), and a collaboration between researchers and students from different universities in different countries (University of Amsterdam in the Netherlands; the School of Health Sciences of the Technical University in Nairobi, Kenya; the School of Nursing of the University of Ghana, Accra, Ghana). The uniqueness of the project is also reflected in the fact that it was community based in the sense that every stage of the research (from putting together the topic list for the interviews and the questions for the paper-pencil survey through reporting the findings back to NGO’s and patient groups) was done in close collaboration with support groups (the Footsteps to Fertility Foundation, Nairobi and the Association of Childless Couples of Ghana, Accra), private fertility clinics (Footsteps to Fertility Clinic, Nairobi; Pentecost Hospital Madina, Accra) and the Walking Egg Foundation. The Walking Egg Foundation is a non-profit organization, aiming to make good quality infertility care more accessible and available for those infertile couples who are at present excluded from treatment. This will be done through a multidisciplinary and global approach (Ombelet, 2013, 2014; www.thewalkingegg.com).

## The studies

In both countries quantitative and qualitative data were collected among visitors and staff of the fertility clinics and members of the fertility support groups. Data collection took place between March and November 2016. Bachelor’s and Master’s students of the above mentioned faculties conducted the research as part of their Master’s thesis (Bilajbegovic, 2016; Koppen, 2016; Oomen, 2016; Van Stiphout, 2016; Kemunto Migiro, in process; Kioko, in process) or assisted in data collection, under supervision of faculty staff. In total 164 survey questionnaires have been completed (79 in Kenya and 85 in Ghana); and 79 in-depth interviews were done with women and men facing fertility problems (42 in Kenya and 37 in Ghana) and 17 with staff and other key informants (10 in Kenya and 7 in Ghana). In addition the students organized focus group discussions with women and men facing fertility problems (2 in Kenya and 1 in Ghana) and conducted observations at clinics and during activities of the support groups. Ethical clearance and research permits were received from review boards of the Amsterdam Institute of Social Science Research of the University of Amsterdam, of National Commission for Science and Technology in Kenya, the Kenyatta National Hospital/University of Nairobi and of the Noguchi Memorial Institute for Medical Research (NMIMR) of the University of Ghana. 

**Figure 2:**
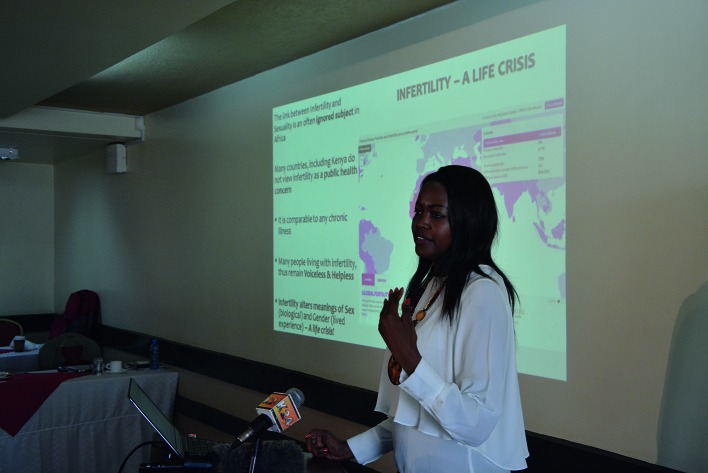
— Dr. Tammary Esho presenting about infertility & sexuality during the workshop for practitioners.

## Dissemination Workshops

After data collection and analysis the next step in the project was the dissemination and discussion of the findings in workshops with relevant stakeholders, including the women and men facing fertility problems themselves, the fertility support groups, policy makers, representatives of ministries of health, NGOs and other professionals in the field of sexual and reproductive health. The aims of the workshops are to increase awareness and knowledge, to validate research findings and to discuss recommendations for the future.

This commentary focuses on the dissemination workshops in Kenya. The dissemination workshops in Ghana will follow in the summer of 2017. On the 2nd and 3rd of December 2016 the first two dissemination workshops have been held in Nairobi, Kenya. One of these workshops was for ‘practitioners’ working in the field of infertility – including medical doctors, nurses, psychologists, counsellors, representatives of patients support group, and researchers and was also attended by the media– and the other one for and with women and men confronted with fertility problems. Both workshops were hosted by the Footsteps to Fertility Foundation.

Based on the data collected in Kenya presentations were given on the infertility situation in Kenya (e.g., prevalence and number of clinics); meaning of parenthood and consequences of infertility; patients’ knowledge and experiences regarding treatment (and use of ART); the importance of sharing and support for people with fertility problems; the role of support groups in practice. Furthermore, additional presentations were given on the following themes (not based on research in Kenya): sexuality issues related to infertility; third party involvement in conception: surrogacy and gamete donation; and new developments in treatments (Ombelet, 2013, 2014; www.thewalkingegg.com). At the end of the workshop we presented and discussed a number of recommendations aiming to improve the lives of men and women facing fertility problems. In the discussion, the audiences - both professionals and patients - to a large extend recognized and agreed with the presented insights and recommendations.

The first recommendation was that tackling stigmatization of infertility is a major issue to counteract the negative images and reactions towards infertile women and men. The audience agreed that the victims should not be blamed and humiliated, but rather supported. The fact that both male and female infertility exist should be widespread, as well as the fact that male infertility is NOT the same as sexual impotence. Public education campaigns have to be put in place to further destigmatize infertility. The public ‘coming out’ of infertile people may be a good means to decrease the stigma and social suffering associated with infertility.

Workshop participants also underlined the importance of engaging religious leaders into destigmatizing infertility and its consequences. However, they warned that not all religious leaders have adequate knowledge about infertility and assisted reproductive technologies (ARTs) and may not always be in favour of the use of these technologies, as this may involve the disposal of embryos, not to speak of using gamete donation and surrogacy. This should be taken into account when seeking collaboration with religious leaders for this purpose.

Next, at all levels in the health system, clinic staff has a major role to play in properly diagnosing both women and men (as our research findings show that this is not always the case) and informing them properly. Information should be realistic and address issues as the available treatment options, potential risks and success rates, in order not to create false hope. Moreover, whenever high tech ARTs are offered, adequate information and counselling should be provided to support women and men in decision making about complex and major choices in infertility treatments, which enables them to carefully weigh the pros and cons of all these options. This is even more important when treatments involve third parties (sperm and egg donation or surrogacy).

The audience, in particular patients, emphasized the importance of adequate professional psychosocial counselling and support - emotionally and practically - to men and women facing fertility problems. Infertility support groups can have an important role in this in terms of providing peer support and information about medical, relational, sexual and emotional aspects of infertility. This is illustrated by one of the women with fertility problems attending the Infertility Workshop in Nairobi:

Sharing your fertility problems with others in the same situation does not resolve your problem, but it does help. Sharing your problem resolves half of it.

Importantly, the unmet needs for ARTs should be addressed: safe and effective IVF should be accessible and affordable to all who need it. Therefore, ‘more affordable’ IVF initiatives - as the one developed by the Walking Egg Foundation (Ombelet, 2013, 2014; www.thewalkingegg.com) - should be strongly supported. At the same time the experiences with and results of this and other new initiatives should be carefully monitored and studied.

Finally, the audience agreed that relevant stakeholders and institutions, including policy makers, local NGOs and international donor agencies working in the field of SRHR, should recognize the importance and impact of infertility. Both in terms of what is at stake for the people involved, and in terms of numbers of people who are affected. They have to be informed about the sort of activities and interventions that can be undertaken, as mentioned above. Strong advocacy is needed: Infertility support groups and professional organizations have an important role to play here. Seeking collaboration with journalists, the mass media and social media to attract the attention of policy makers might be an effective strategy and has been proven to be successful in other fields (Chetley et al., 2007). In addition, continuing research - clinical, epidemiological and social science studies – is crucial to inform, monitor and build further evidence regarding the proposed recommendation. We all have a role to play. This unique workshop in Kenya – and other workshops are planned in Ghana – showed the urgency and willingness to address the social suffering of infertility. Only by working together we can make a difference.

**Figure 3:**
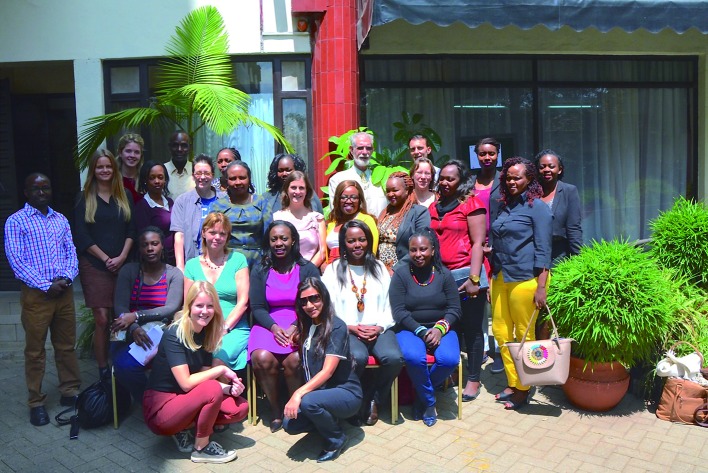
— Attendants Workshop for practitioners.
